# Supplementary material for: Patterns of Intron Gain and Loss in Fungi
Source: PLoS Biol. 2004 Nov 30;2(12):e422. doi: 10.1371/journal.pbio.0020422 (PMC532390; doi:10.1371/journal.pbio.0020422)
Supplement: Table S1 — Also available at http://genes.mit.edu/NielsenEtAl/. (4.3 MB ZIP). [file pbio.0020422.st001.zip › NielsenEtAl/html/193.html]

AN7540.1.NCU07380.1.MG00341.1.FG01314.1


```
 CLUSTAL W (1.82) Multiple Sequence Alignments - Introns Inserted


Sequence 1: NCU07380.1	591 aa
Sequence 2: FG01314.1	576 aa
Sequence 3: MG00341.1	573 aa
Sequence 4: AN7540.1	586 aa
Alignment Length: 606 aa
Number Identitical Residues: 320 aa
Alignment Score (without introns) 15182


MG00341.1 	---MSQSLA------DIIAALP-DGDGWGPAVTTELTLNGVPYAPFSKGDKLGRMADWTD
NCU07380.1	---MTNPTPGLSGLVDLIDSLPDLGATWGPAITTETTLNGVPYAPYSKGDKLGRMADWTA
FG01314.1 	---MTDSLD----YINICESCP-AGDGWGPSVTSETTLNGVPYAPFSKGDKLGRMADWTA
AN7540.1  	MAPIS--------IADIVAALP-AEDTWGPVTSADNMLDGVPYAPFSKGDKLGRMADWTA
          	 :.::          ::  : *     ***  :::  *:******:************* 

MG00341.1 	-GKD-RDGRGGRQQYNRNFRD1QQVYGAGTASLFSAPQAEDESTFSVVSNVRDSTKTRFG
NCU07380.1	DSKDGRDGRGGRQQYNRNFRD1QQIYGAGSTSIFAPPVAEDESSFSVVSNVKDTGKTRFG
FG01314.1 	EGKD-RD-RGGRAQYNRNFRD1QQVYGSSHAITFNAPPAEDESTFSVVSNVRDSTKSRYG
AN7540.1  	DSKD-RD-RGGRQAYNRNYRD1QQVYGAGSSSLFAVQVAEDESTFSVVDNTRTSAKRTFG
          	..** ** ****  ****:** **:**:. :  *    *****:****.*.: : *  :*

MG00341.1 	RG-AVFTRGGRGQRGA---RGTERGGRAQLSR-GRGGQYGGG---YDRGGRSAAGGRGGR
NCU07380.1	RG-AVFTRG-RGQRGGAGAQAGRGGGRQTFQRTGRGGQQYGGGYDGGRGGRQNTG-ARGR
FG01314.1 	RG-AVFTRG-RGQRGG---QAGARGGRTTLQ---RGGANAGGRQGYDRGGRNNAG-RGGR
AN7540.1  	RGGGTVFRG-RAQRGG----AGQRGGRAGFQRVGAGRGQGGDRYYDNRGARGNRG----R
          	**.... ** *.***.    .   ***  :.  . *    *.    .**.*   *    *

MG00341.1 	RFGWKDYDKPARNRDASINVKADWSLLEEIDFNRLGKLNLDADEGEDVDSYGFVHYYDRS
NCU07380.1	RFGWKDYDKPQRNRDASINIKGDWKLIEEIDYNRLSKLNLETDEGEDVDDYGFLYAYDRS
FG01314.1 	RFGWKDYDKPARNRDASINVKAEWKLLEEIDFNRLAKLNLDTDEGEDIDDYGFLYPYDRS
AN7540.1  	RFGWKDYDKPQRTREPSVNIRPDWSMLEEVDFNRLSKLNLQAPEGEDVDTYGFLYYYDRS
          	********** *.*:.*:*:: :*.::**:*:***.****:: ****:* ***:: ****

MG00341.1 	YDKPAVKSAERKLNPIDRAAYNVTTSLDPVIQELAEKDEATIFATDNILSTLMCAPRSVY
NCU07380.1	YDKPVVKNAERRLAAIDRAAYNVTTSSDPVIQELAEKDEATIFATDNILSMLMCAPRSVA
FG01314.1 	YDKQPVKGAEKKLTPIDRAAYNVTTSSDPVIQELAEKDEATIFATDSILSMLMCSPRSVY
AN7540.1  	YDKAPVKNAERRLQALDRAAYNVTTSQDPVIQELAEKNQATIFATSDILSMLMCAPRSVY
          	***  **.**::* .:********** **********::******..*** ***:**** 

MG00341.1 	PWDIVIVKQGSKVYFDKRDNAALDMVTVNENAVDAPLEASE--GSKDSINQPSALAEEAT
NCU07380.1	PWDIVISRQGNKIFFDKRDNAALDLVTVNENAADAPLDASE--GSKDGINQPHALAEEAT
FG01314.1 	PWDIVISRQGNKIFLDKRDNAALDMVTVNENAADAPLDAAD--GSKDTINQPNALAEEAT
AN7540.1  	SWDIVIVHQGNKIYFDKREGASLDLVTVNENAADAPLEVAESSGKQESINTPSALALEAT
          	.***** :**.*:::***:.*:**:*******.****:.::::*.:: ** * *** ***

MG00341.1 	YINHNFVNQVVIENESQKVDMEHENPFYNAAEETEPPASKAYKYRKFDLST-NDEGPVYL
NCU07380.1	FINHNFANQVVVENESEKVNMAHENPFYNASEENVPPASKAYKYRRFDLST-SEEESTFL
FG01314.1 	YINHNFTNQVVVENESAKVNMAHGNPFYNSSEDTDPPASKAYKYRRFDLST-NEEEPVHL
AN7540.1  	FINHNFALQTVVEANNAKVDFNHPNPFYNAEEETEPLASKGYKYRRFDLSLESDEEPLNM
          	:*****. *.*:* :. **:: * *****: *:. * ***.****:**** ..:* .  :

MG00341.1 	VVRTELDAVQKNSNNGEDQFLTVRALNEFDNKAQGSGGALDWRSKLVSQRGAVVATEMKN
NCU07380.1	VVRTEIDAVQKNATDGKNQLVTVHALNEWDSKAQGAGGALDWRTKLASQRGAVVATEMKN
FG01314.1 	IVRTEVDAVQKNAISGEDQHISIHALNEFDSKAQGSGGALDWRSKLVTQRGAVVATEMKN
AN7540.1  	IVRTEVDALVKNPVNGEDQQLVVKALNEFDSKAQGSGGSLDWRSKLYSQRGAVVATEMKN
          	:****:**: **. .*::* : ::****:*.****:**:****:** :************

MG00341.1 	NSCKLAKWTVQSILAKADVLKLG2FVSRANPKSNDKHVILGVIGWKPKDFANQMNLHLSN
NCU07380.1	NSCKLARWTVQSILAKADVMKLG2FVSRVNPKVNDKHVILGVVGWKPKDFANQMNLQLSN
FG01314.1 	NSCKLARWTVQSILASADVMKLG2FVSRVTPRSNDKHVVLGVIGWKPRDFANQMNLSLNN
AN7540.1  	NSCKLARWTTQAILAKADGMKLG~FVSRANPRSAAAHVVLGVAGYKPREFAAQMNLNLGN
          	******:**.*:***.** :*** ****..*:    **:*** *:**::** **** *.*

MG00341.1 	GWGIVRTIADMCLKR--EDG-----KYVLVKDPNKTILRLYEVPAGGLDEEEDNGDLGQE
NCU07380.1	GWGIVRTIADMCLKSESEDA-----KFVLVKDPNKNILRLYEVPAGSLDEEAEELDQVEE
FG01314.1 	GWGIVRTIADMILSSSTQDA-----KFVLVKDPNKSILRLYEVPTGSFDDDDEE--EVEE
AN7540.1  	GWGIVRTIVDRIRALDADEEEDKVTKYILVKDPNRPVLRLYSVPANAFEEDDEAAEEEQE
          	********.*     .::: ... :*::******: :****.**:..:::: :  .  :*

MG00341.1 	EDDEE---
NCU07380.1	DGEEAEE-
FG01314.1 	PQGEEEQ-
AN7540.1  	AKGEVEEA
          	   * ..:
```
